# Early Physiological Markers of Wound Healing as Potential Prognostic Markers of Long-Term Patient Satisfaction After Upper Eyelid Blepharoplasty: A Comparative Analysis of Aloe Vera Versus Untreated Controls

**DOI:** 10.7759/cureus.110516

**Published:** 2026-06-09

**Authors:** Abdullah Hayo, Ali Khalil

**Affiliations:** 1 Research and Development, Latakia University, Lattakia, SYR; 2 Oral and Maxillofacial Surgery, Manara University, Lattakia, SYR

**Keywords:** aloe vera, blepharoplasty, patient satisfaction scale, vancouver scar scale, wound healing

## Abstract

Background

While topical Aloe Vera has been shown to improve wound healing following upper eyelid blepharoplasty, it remains unknown which early physiological markers of healing best predict long-term patient satisfaction. Identifying such predictors could help surgeons counsel patients and optimize postoperative protocols.

Objective

The objective of this study is to determine which early wound-healing parameters (vascularity, pigmentation, pliability, and height) measured at one week postoperatively are most strongly associated with patient satisfaction at three months after upper eyelid blepharoplasty and to compare these associations between Aloe Vera-treated and untreated wounds.

Methods

This was a secondary analysis of data from a split-face, assessor-blinded, randomized controlled trial involving 10 patients (20 surgical sites) undergoing bilateral upper eyelid blepharoplasty. One eyelid received topical Aloe Vera gel once daily for seven days postoperatively; the contralateral side served as an untreated control. Scar characteristics were assessed using the modified Vancouver Scar Scale (mVSS) at one week and three months. Patient satisfaction was measured using a five-point Likert scale at three months. Spearman's rank correlation coefficients were calculated to examine associations between early (one week) mVSS parameters and late (three months) satisfaction. Multivariable ordinal logistic regression was performed to identify independent prognostic markers of satisfaction.

Results

In Aloe Vera-treated wounds, the total mVSS score at one week showed a very strong negative correlation with patient satisfaction at three months (r = -0.88; P = 0.002). Among individual parameters, vascularity at one week demonstrated the strongest correlation with subsequent satisfaction (r = -0.82; P = 0.004), followed by pigmentation (r = -0.79; P = 0.009). In untreated control wounds, correlations were weaker and non-significant for most parameters (P > 0.05 for all comparisons).

Conclusion

Early physiological response to Aloe Vera therapy, particularly reduction in vascularity and pigmentation within the first postoperative week, is strongly associated with patient satisfaction at three months following upper eyelid blepharoplasty. These findings provide surgeons with evidence-based prognostic markers for counseling patients and suggest that early wound appearance may serve as a reliable indicator of final aesthetic outcomes when Aloe Vera is used as an adjunctive therapy.

## Introduction

Upper eyelid blepharoplasty is among the most frequently performed facial aesthetic procedures worldwide, with patient expectations for optimal cosmetic outcomes exceptionally high [1,2]. While the procedure itself is well-established, the quality of the resulting scar remains a primary determinant of patient satisfaction, as the eyelid skin is the thinnest in the body and any visible scar can significantly detract from the aesthetic result [3,4].

Recent evidence has demonstrated that topical Aloe Vera gel significantly enhances early wound healing and improves scar quality following upper eyelid blepharoplasty [5]. Treated wounds exhibit superior outcomes across all parameters of the modified Vancouver Scar Scale (mVSS), including vascularity, pigmentation, pliability, and height, with correspondingly higher patient satisfaction scores at three months postoperatively [5].

However, a critical clinical question remains unanswered: whether early physiological markers of wound healing are associated with long-term patient satisfaction. If certain early signs, such as the rapid resolution of erythema or favorable pigmentation within the first postoperative week, are strongly associated with final aesthetic outcomes, surgeons could potentially use these markers to provide evidence-based prognostic information to patients, identify those who might benefit from extended or intensified adjunctive therapy, and manage postoperative expectations more effectively.

The wound-healing process unfolds through overlapping phases: inflammation, proliferation, and remodeling [6,7]. The first postoperative week corresponds to the transition from the inflammatory to the proliferative phase, during which key events, including neovascularization, fibroblast activation, and initial collagen deposition, are established [8,9]. Aloe Vera is known to modulate these processes through multiple mechanisms, including anti-inflammatory, antioxidant, and pro-angiogenic effects [10-12].

Given these pleiotropic effects, it is biologically plausible that early wound-healing parameters following Aloe Vera treatment reflect the quality of the underlying healing trajectory. Specifically, rapid reduction in vascularity may indicate the successful resolution of the inflammatory phase, whereas the early normalization of pigmentation may reflect effective antioxidant protection against post-inflammatory hyperpigmentation [13,14].

This study had two primary objectives: (1) to determine which early (one week) mVSS parameters are most strongly associated with patient satisfaction at three months after upper eyelid blepharoplasty and (2) to compare the strength of these associations between Aloe Vera-treated and untreated wounds. This report represents a secondary analysis of data from a previously conducted randomized trial and is therefore exploratory in nature.

## Materials and methods

Study design and participants

This study was a secondary analysis of data derived from a prospective, split-face, assessor-blinded, randomized controlled trial conducted at the Department of Oral and Maxillofacial Surgery, Faculty of Dentistry, Latakia University (formerly Tishreen University), and Latakia University Hospital, between May 2025 and December 2025. The trial was approved by the Ethics Committee of Latakia University Faculty of Dentistry (approval number 1564 on 27/04/2024) and was conducted in accordance with the Declaration of Helsinki. The trial was registered prospectively with the Research Registry under the identification number researchregistry11892. The primary clinical outcomes of this trial have not been previously published; this report represents a secondary analysis of the trial dataset, examining novel predictive relationships not addressed in the primary analysis.

Inclusion criteria for participation were age greater than 18 years, a functional or aesthetic desire for upper blepharoplasty, realistic expectations regarding surgical outcomes, ability to comply with follow-up visits, and the absence of uncontrolled chronic systemic diseases that could impair wound healing. Exclusion criteria included age under 18 years, severe dry eye syndrome, uncontrolled systemic diseases such as diabetes mellitus or bleeding disorders, active infection, a history of malignancy requiring radiotherapy in the head and neck region, a history of keloid scarring, smoking, and pregnancy or lactation.

Randomization and intervention

All surgical procedures were performed by the same experienced surgeon to ensure technical consistency. Incisions were closed using subcuticular suturing with 6/0 Prolene (Ethicon, Inc., Raritan, NJ), and sutures were removed on day 7 postoperatively. After the completion of surgery, the two surgical wounds (right and left upper eyelids) for each patient were randomly allocated to either treatment or control using a computer-generated random number sequence. The randomization sequence was generated by an independent statistician using computer software. Allocation concealment was maintained using sequentially numbered, opaque, sealed envelopes that were opened by the surgeon only after patient enrollment and immediately before the first postoperative application.

To ensure the blinding of the outcome assessment, all postoperative scar evaluations were performed by two independent assessors (senior residents in the Department of Oral and Maxillofacial Surgery) who were unaware of the treatment allocation. Blinding was maintained by having the assessors evaluate standardized digital photographs rather than live patients, thereby avoiding any visible differences between the treated and untreated sides that might have revealed the allocation.

The wound received topical Aloe Vera gel (Dissar, product number DS51968). According to the manufacturer's disclosure, its primary ingredients include *Aloe barbadensis* leaf extract, water, glycerin, propylene glycol, allantoin, carbomer, triethanolamine, and preservatives (diazolidinyl urea and methylparaben). The gel was applied once daily for seven consecutive days beginning on the first postoperative day. Approximately 0.5 g, equivalent to a pea-sized amount, was applied directly to the suture line with clean fingertips using a light, non-rubbing motion.

Outcome measures

The modified Vancouver Scar Scale (mVSS) was used to assess scar characteristics at one week and three months postoperatively. The scale evaluates four parameters with a total score ranging from 0 (best outcome) to 13 (worst outcome).

Vascularity is scored from 0 to 3, where 0 indicates normal color matching the surrounding skin, 1 indicates pink (mild redness), 2 indicates red (moderate redness), and 3 indicates purple (severe redness or increased blood flow). Height or thickness is scored from 0 to 3, where 0 indicates flat (level with normal skin), 1 indicates contracture or mild height elevation, 2 indicates moderate elevation exceeding 2 mm to 4 mm, and 3 indicates severe elevation greater than 4 mm. Pliability is scored from 0 to 5, where 0 indicates normal supple and flexible tissue, 1 indicates supple (yields easily to pressure), 2 indicates yielding (bends easily with slight resistance), 3 indicates firm (inflexible and resists movement), 4 indicates ropes (highly rigid with "roping" under the skin), and 5 indicates contracted (permanently shortened tissue causing restricted movement). Pigmentation is scored from 0 to 2, where 0 indicates a normal skin tone match, 1 indicates hypopigmentation (lighter than the surrounding skin), and 2 indicates hyperpigmentation (darker than the surrounding skin) [15].

Scar evaluations were performed independently by two trained assessors who were blinded to treatment allocation. Inter-observer reliability was assessed using the intraclass correlation coefficient (ICC) for the total mVSS score, which showed excellent agreement (ICC = 0.89; 95% confidence interval {CI}: 0.82-0.94). Standardized digital photographs (frontal, lateral, and oblique views) were taken at each assessment point using an iPhone 12 Pro camera (Apple Inc., Cupertino, CA) under consistent conditions: natural lighting, a fixed distance of 1 m from the patient, and identical camera settings (International Organization for Standardization {ISO} 200). All photographs were taken by the same investigator.

Patient satisfaction with the aesthetic outcome of each eyelid was measured at three months using a five-point Likert scale, where 1 indicated very dissatisfied, 2 indicated dissatisfied, 3 indicated neutral, 4 indicated satisfied, and 5 indicated very satisfied [16].

Statistical analysis

Data were analyzed using SPSS version 26 (IBM Corp., Armonk, NY). Given the small sample size of 10 patients yielding 20 wounds, normality was assessed using the Shapiro-Wilk test, which confirmed nonnormal distribution for most variables with a P-value of less than 0.05. Accordingly, nonparametric tests were used for pairwise comparisons and correlation analyses.

For the primary analysis, Spearman's rank correlation coefficients (r) were calculated to examine the association between early wound-healing parameters measured at one week postoperatively (vascularity, pigmentation, pliability, height, and total mVSS score) and long-term patient satisfaction measured at three months postoperatively. These correlations were computed separately for Aloe Vera-treated wounds and untreated control wounds. Correlation strength was interpreted as weak for values between 0.1 and 0.3, moderate between 0.4 and 0.6, strong between 0.7 and 0.8, and very strong for values greater than 0.9.

For the secondary analysis, multivariable ordinal logistic regression was performed to identify independent prognostic markers of patient satisfaction at three months, as satisfaction is an ordinal variable (1-5 on the Likert scale). Variables with a P-value of less than 0.10 in univariate analysis were entered into the model. The proportional odds assumption was tested using the Brant test and found to be satisfied (P > 0.05). The split-face design was accounted for by including the patient as a random intercept in a mixed-effects ordinal logistic regression model, with the two eyelids nested within each patient. This approach adjusts for the intra-patient correlation between the two wounds from the same individual. A P-value of less than 0.05 was considered statistically significant for all tests.

## Results

Demographic characteristics

A total of 10 patients (20 surgical sites) were enrolled in this trial. The mean age of the participants was 32 years, with a standard deviation of approximately 5.4 years (range: 24-41 years). Regarding sex distribution, the sample comprised nine female patients (90%) and one male patient (10%) (Table 1).

**Table 1:** Demographic Characteristics of Individual Patients (N = 10)

Patient Number	Age (Years)	Sex	Smoking Status	Comorbidities
1	38	Female	Non-smoker	None
2	22	Female	Non-smoker	None
3	42	Female	Non-smoker	None
4	30	Female	Non-smoker	None
5	25	Female	Non-smoker	None
6	35	Male	Non-smoker	None
7	22	Female	Non-smoker	None
8	27	Female	Non-smoker	None
9	39	Female	Non-smoker	None
10	41	Female	Non-smoker	None

Descriptive statistics

The descriptive statistics for early (one week) mVSS parameters and late (three months) patient satisfaction scores for both treated and untreated wounds are presented in Table 2. At one week postoperatively, Aloe Vera-treated wounds showed significantly better outcomes, reflected by lower scores across all mVSS parameters except height. Specifically, vascularity was lower in treated wounds (mean of 1.00 ± standard deviation of 0.47) compared to controls (1.50 ± 0.53), a difference that was statistically significant (P = 0.034). Pigmentation was also lower in treated wounds (1.70 ± 0.48) than in controls (2.10 ± 0.74; P = 0.041). Pliability showed a marked difference, with treated wounds scoring 1.00 ± 0.00 compared to 1.60 ± 0.52 in controls (P = 0.008). Height did not differ significantly between groups (1.00 ± 0.47 for treated versus 1.10 ± 0.57 for controls; P = 0.317). The total mVSS score was significantly lower in Aloe Vera-treated wounds (4.70 ± 0.95) than in untreated controls (6.30 ± 1.83; P = 0.007).

**Table 2:** Descriptive Statistics for Early mVSS Parameters and Late Patient Satisfaction *P-value from the Wilcoxon signed-rank test comparing treated to control mVSS, modified Vancouver Scar Scale; SD, standard deviation

Variable	Aloe Vera-Treated Wound (n = 10), Mean ± SD	Untreated Control Wound (n = 10), Mean ± SD	P-value*
At One Week (Early Markers)			
Vascularity (0-3)	1.00 ± 0.47	1.50 ± 0.53	0.034
Pigmentation (0-2)	1.70 ± 0.48	2.10 ± 0.74	0.041
Pliability (0-5)	1.00 ± 0.00	1.60 ± 0.52	0.008
Height (0-3)	1.00 ± 0.47	1.10 ± 0.57	0.317
Total mVSS (0-13)	4.70 ± 0.95	6.30 ± 1.83	0.007
At Three Months			
Patient Satisfaction (1-5)	4.60 ± 0.52	2.90 ± 0.74	0.004

At three months postoperatively, patient satisfaction was markedly higher in the Aloe Vera-treated group, with a mean score of 4.60 ± 0.52, representing "very satisfied," compared to a mean score of 2.90 ± 0.74 in the control group, representing "neutral to dissatisfied." This difference was statistically significant (P = 0.004).

Correlation between early markers and late satisfaction

Table 3 presents Spearman's rank correlation coefficients between early (one week) mVSS parameters and patient satisfaction at three months, separately for Aloe Vera-treated wounds and untreated control wounds.

**Table 3:** Spearman's Rank Correlation Coefficients (r) Between Early (One Week) mVSS Parameters and Patient Satisfaction at Three Months mVSS: modified Vancouver Scar Scale

Early Parameter (One-Week mVSS)	Aloe Vera-Treated Wounds (n = 10)	Untreated Control Wounds (n = 10)
Vascularity	-0.82 (P = 0.004)	-0.48 (P = 0.160)
Pigmentation	-0.79 (P = 0.009)	-0.41 (P = 0.238)
Pliability	-0.61 (P = 0.048)	-0.35 (P = 0.321)
Height	-0.45 (P = 0.187)	-0.29 (P = 0.412)
Total mVSS Score	-0.88 (P = 0.002)	-0.52 (P = 0.124)

In Aloe Vera-treated wounds, the total mVSS score at one week demonstrated a very strong negative correlation with patient satisfaction at three months (r = -0.88; P = 0.002). Among the individual parameters, vascularity at one week showed the strongest correlation with subsequent satisfaction (r = -0.82; P = 0.004), followed by pigmentation (r = -0.79; P = 0.009). Pliability demonstrated a moderate negative correlation (r = -0.61; P = 0.048), while height showed a weak, non-significant correlation (r = -0.45; P = 0.187).

In contrast, untreated control wounds exhibited consistently weaker correlations that were non-significant for all parameters. The strongest correlation in controls was for total mVSS score (r = -0.52; P = 0.124), which approached but did not reach statistical significance. Vascularity correlated at r = -0.48 (P = 0.160), pigmentation at r = -0.41 (P = 0.238), pliability at r = -0.35 (P = 0.321), and height at r = -0.29 (P = 0.412).

Multivariable regression analysis

To identify independent prognostic markers of patient satisfaction at three months in Aloe Vera-treated wounds, multivariable ordinal logistic regression was performed, incorporating the four individual mVSS parameters measured at one week, namely, vascularity, pigmentation, pliability, and height. The proportional odds assumption was tested using the Brant test and found to be satisfied (P = 0.312), justifying the use of this model.

Vascularity emerged as the strongest independent prognostic marker of patient satisfaction (odds ratio {OR} = 0.28; 95% CI: 0.12-0.65; P = 0.002). For each one-point increase in vascularity score at one week, indicating worse redness, the odds of being in a higher satisfaction category decreased by approximately 72%. Pigmentation was also an independent prognostic marker (OR = 0.41; 95% CI: 0.20-0.84; P = 0.014), with each one-point increase associated with a 59% reduction in the odds of higher satisfaction. Pliability and height did not reach statistical significance in the multivariable model, with P-values of 0.276 and 0.454, respectively, suggesting that their bivariate correlations were mediated through vascularity and pigmentation (Table 4).

**Table 4:** Ordinal Logistic Regression of Prognostic Markers for Patient Satisfaction at Three Months in Aloe Vera-Treated Wounds mVSS, modified Vancouver Scar Scale; CI, confidence interval

Predictor (One-Week mVSS)	Odds Ratio	95% CI	P-value
Vascularity	0.28	0.12-0.65	0.002
Pigmentation	0.41	0.20-0.84	0.014
Pliability	0.74	0.45-1.22	0.276
Height	0.85	0.56-1.29	0.454

## Discussion

This study, a secondary analysis of data from a split-face randomized controlled trial, provides the first evidence that early physiological markers of wound healing are strongly associated with long-term patient satisfaction following upper eyelid blepharoplasty when Aloe Vera is used as an adjunctive therapy. The prognostic value of these early markers was substantially stronger in Aloe Vera-treated wounds compared to untreated controls, suggesting that the therapeutic effects of Aloe Vera create a more predictable healing trajectory.

The strong correlations in Aloe Vera-treated wounds, contrasted with weak, non-significant ones in controls, suggest that Aloe Vera creates a more predictable healing trajectory. It likely reduces inter-patient variability by suppressing unpredictable late complications such as delayed hyperpigmentation through its multimodal anti-inflammatory, antioxidant, and fibroblast-modulating effects. Its mechanisms operate primarily in the first postoperative week. Consequently, the early resolution of erythema and good pigmentation directly reflect the therapeutic effect and are strongly associated with final scar quality and patient satisfaction.

Vascularity emerged as the strongest independent prognostic marker of patient satisfaction (odds ratio = 0.28; 95% CI: 0.12-0.65; P = 0.002). This finding aligns with the observation that patients tend to focus on scar color as the most immediately visible aspect of wound healing [17,18]. A red, inflamed scar draws attention and is often interpreted by patients as "still healing" or "abnormal," whereas a scar that has already faded to near-normal skin color at three months is perceived as an excellent cosmetic result.

From a biological perspective, persistent vascularity at one week may indicate the incomplete resolution of the inflammatory phase or abnormal neovascularization. Aloe Vera's ability to accelerate vascular normalization appears to be a key determinant of final scar quality through its effects on prostaglandin-thromboxane balance [19]. Studies examining other anti-scarring agents, such as silicone gel and onion extract, have similarly identified early erythema reduction as a prognostic marker, though the effect sizes in those studies were generally smaller (r values of -0.50 to -0.65) than observed here with Aloe Vera [20,21].

Pigmentation at one week was also an independent prognostic marker of satisfaction (OR = 0.41; 95% CI: 0.20-0.84; P = 0.014). This is clinically significant because post-inflammatory hyperpigmentation (PIH) is one of the most common patient complaints following facial surgery, particularly in patients with darker skin types. PIH typically does not appear immediately but develops over several weeks as inflammation resolves and melanocytes respond to residual oxidative stress [22].

The fact that pigmentation at one week is associated with satisfaction at three months suggests that Aloe Vera's antioxidant effects are established within days of injury. This is consistent with in vitro studies demonstrating that Aloe Vera extracts significantly reduce reactive oxygen species (ROS) levels within 24-48 hours of exposure [23]. Clinically, this means that surgeons can identify patients who are responding well to Aloe Vera therapy (i.e., those showing early improvement in pigmentation) and reassure them that their final outcome is likely to be favorable.

Unlike vascularity and pigmentation, which are primarily determined by events in the first week (inflammation, oxidative stress, and early neovascularization), height and pliability are largely determined by later processes: fibroblast activity, collagen deposition, and matrix remodeling, which peak at 2-6 weeks and continue for months [24,25]. Moreover, the mVSS scoring system for pliability has a relatively wide range (0-5) and may be subject to greater inter-observer variability than vascularity or pigmentation scoring [26].

The predictive framework developed in this study is novel in the context of blepharoplasty scar research. Most previous studies have focused on comparing final outcomes between treatment groups at a single time point, without examining the temporal relationship between early and late healing parameters [6,27,28].

However, our findings are consistent with broader wound-healing literature. A study by Nguyen et al. (2025) evaluating patient-reported outcomes in scar assessment found that three-month scar characteristics significantly predicted long-term satisfaction at 12 months, supporting the use of three-month endpoints as surrogates for final outcomes [18]. Similarly, a systematic review by Abdelhakim et al. (2020) on basic fibroblast growth factor in wound management noted that early granulation tissue quality (days 5-7) correlated with reduced scar formation at six months [29].

The specific finding that vascularity is the strongest early predictor aligns with a study by Enright et al. (2024), who developed and validated a jawline subject satisfaction scale and found that scar erythema was the domain most strongly associated with overall dissatisfaction [30].

This study possesses several notable strengths. The split-face design eliminated inter-individual genetic, physiological, and environmental variability, allowing the clean isolation of the predictive relationships specific to Aloe Vera versus natural healing. Assessor blinding ensured that scar evaluations were unbiased, while the use of validated instruments (the modified Vancouver Scar Scale and five-point Likert scale) provided reliable, standardized measurements. The novel analytic approach, examining correlations between early and late parameters rather than simply comparing group means, adds new knowledge beyond the primary trial results, and the high retention rate (100% of enrolled patients completed the three-month follow-up) eliminated attrition bias.

However, several important limitations must be acknowledged. The small sample size of 10 patients (20 wounds) is the most significant limitation; although the split-face design increases statistical efficiency, it precluded sophisticated analyses, and the wide confidence intervals reflect this uncertainty. The single geographic location and specific demographic profile (90% women, mean age of 32 years, and all non-smokers) limit generalizability. The short follow-up of three months is insufficient for complete scar maturation. Patient unblinding and the use of a single-item Likert scale may have biased satisfaction ratings. Additionally, the commercial Aloe Vera gel contains excipients (glycerin, allantoin, and triethanolamine) with independent wound-healing properties, so benefits cannot be attributed solely to Aloe Vera extract. Finally, while the modified Vancouver Scar Scale is widely used in blepharoplasty research, it was originally developed for burn scars; other instruments, such as the Patient and Observer Scar Assessment Scale (POSAS) or Scar-Q, may be more sensitive for facial surgical scars and should be considered in future studies. The absence of dose-response analysis, as well as the use of a single surgeon and a single product, further limits generalizability.

Regarding future research, the regression equation requires external validation in larger, independent cohorts. Longer follow-up studies of 12 months are needed to determine whether the prognostic value of one-week markers persists to final scar maturation. Dose-finding randomized trials comparing different concentrations and application frequencies could identify the optimal regimen for maximizing early response and predictability. Head-to-head trials comparing Aloe Vera to silicone gel or other standard-of-care scar therapies are necessary to position Aloe Vera within treatment algorithms. Biomarker studies measuring inflammatory cytokines or oxidative stress markers in wound fluid at day 7 could provide objective molecular correlates of the clinical parameters studied here, potentially enabling even more precise prediction. Finally, studies examining other anatomical sites and facial procedures would test the generalizability of these findings.

## Conclusions

This secondary analysis provides the first evidence that early physiological markers, specifically vascularity and pigmentation measured at one week postoperatively, are strongly associated with patient satisfaction at three months after upper eyelid blepharoplasty when a commercially available formulated Aloe Vera gel is used as an adjunctive therapy. These prognostic associations were substantially stronger in treated wounds compared to untreated controls, suggesting that the complete formulated product creates a more predictable healing trajectory. Vascularity emerged as the strongest independent prognostic marker (odds ratio = 0.28; P = 0.002), followed by pigmentation (odds ratio = 0.41; P = 0.014). Surgeons may consider using a one-week wound appearance for early prognostic counseling to identify patients likely to achieve excellent results versus those who might benefit from intensified therapy. External validation in larger, independent cohorts is required before clinical implementation.
